# Variational mode decomposition-based EEG analysis for the classification of disorders of consciousness

**DOI:** 10.3389/fnins.2024.1340528

**Published:** 2024-02-06

**Authors:** Sreelakshmi Raveendran, Raghavendra Kenchaiah, Santhos Kumar, Jayakrushna Sahoo, M. K. Farsana, Ravindranadh Chowdary Mundlamuri, Sonia Bansal, V. S. Binu, A. G. Ramakrishnan, Subasree Ramakrishnan, S. Kala

**Affiliations:** ^1^Department of Electronics and Communication Engineering, Indian Institute of Information Technology, Kottayam, Kerala, India; ^2^Department of Neurology, NIMHANS, Bangalore, Karnataka, India; ^3^Department of Computer Science and Engineering, Indian Institute of Information Technology, Kottayam, Kerala, India; ^4^Department of Neuroanaesthesia and Neurocritical Care, NIMHANS, Bangalore, Karnataka, India; ^5^Department of Biostatistics, NIMHANS, Bangalore, Karnataka, India; ^6^Department of Electrical Engineering and Centre for Neuroscience, Indian Institute of Science, Bangalore, Karnataka, India

**Keywords:** variational mode decomposition, disorders of consciousness, resting-state EEG, ensemble bagged tree, patient care, resource management, treatment options and rehabilitation strategies

## Abstract

Aberrant alterations in any of the two dimensions of consciousness, namely awareness and arousal, can lead to the emergence of disorders of consciousness (DOC). The development of DOC may arise from more severe or targeted lesions in the brain, resulting in widespread functional abnormalities. However, when it comes to classifying patients with disorders of consciousness, particularly utilizing resting-state electroencephalogram (EEG) signals through machine learning methods, several challenges surface. The non-stationarity and intricacy of EEG data present obstacles in understanding neuronal activities and achieving precise classification. To address these challenges, this study proposes variational mode decomposition (VMD) of EEG before feature extraction along with machine learning models. By decomposing preprocessed EEG signals into specified modes using VMD, features such as sample entropy, spectral entropy, kurtosis, and skewness are extracted across these modes. The study compares the performance of the features extracted from VMD-based approach with the frequency band-based approach and also the approach with features extracted from raw-EEG. The classification process involves binary classification between unresponsive wakefulness syndrome (UWS) and the minimally conscious state (MCS), as well as multi-class classification (coma vs. UWS vs. MCS). Kruskal-Wallis test was applied to determine the statistical significance of the features and features with a significance of *p* < 0.05 were chosen for a second round of classification experiments. Results indicate that the VMD-based features outperform the features of other two approaches, with the ensemble bagged tree (EBT) achieving the highest accuracy of 80.5% for multi-class classification (the best in the literature) and 86.7% for binary classification. This approach underscores the potential of integrating advanced signal processing techniques and machine learning in improving the classification of patients with disorders of consciousness, thereby enhancing patient care and facilitating informed treatment decision-making.

## 1 Introduction

Disorders of consciousness (DOC) encompass a broad range of conditions that involve significant impairments in the level of arousal and awareness (Altıntop et al., 2022). These conditions pose complex challenges for individuals, their families, and healthcare providers. Understanding the various DOC categories and their characteristics is essential for appropriate treatment, and prognostic assessment (Schnakers, 2020; Duszyk-Bogorodzka et al., 2022). One of the most well-known DOC categories is coma (Alnagger et al., 2023). Coma represents a deep unconscious state where individuals are unresponsive and unaware of their surroundings with usually closed eyes and with no evidence of awareness or consciousness (Kondziella et al., 2020). It can result from severe traumatic brain injury, stroke, brain hemorrhage, or other causes that disrupt normal brain function and can be typically defined by the absence of both wakefulness and purposeful responses to stimuli (Alnagger et al., 2023). Unresponsive wakefulness syndrome (UWS), formerly referred to as vegetative state, is another significant DOC category (Morris et al., 2022). In UWS, individuals may exhibit sleep-wake cycles, open their eyes, and have basic physiological functions (Gervais et al., 2023). However, there is a profound lack of awareness, with no signs of purposeful or meaningful interaction with the environment (Morris et al., 2022). The third category of DOC, namely minimally conscious state (MCS) is characterized by individuals displaying a degree of awareness, though limited, and inconsistent wakefulness (Thibaut et al., 2020). Individuals in MCS may show occasional purposeful behavior or response to stimuli that go beyond reflexive or automatic actions (Gervais et al., 2023). These responses may include following simple commands, gesturing, or exhibiting emotional expressions. However, the level of consciousness and awareness in MCS can fluctuate, making it a challenging state to assess accurately (Gervais et al., 2023).

Categorizing patients with disorders of consciousness across the spectrum involves the utilization of behavioral assessment methods, with the Coma Recovery Scale-Revised (CRS-R) serving as the primary tool for evaluating and scoring individuals (Coleman et al., 2009). This comprehensive scale assesses visual, auditory, motor, oromotor, and communication responses, enabling clinicians to distinguish between different states of consciousness (Majerus et al., 2005). The CRS-R provides a detailed insight into a patient's level of consciousness and recovery potential by systematically evaluating specific responses and behaviors. In addition to the CRS-R, established neurobehavioral assessment tools tailored for DoC patients include the Glasgow coma scale (GCS), Western Head Injury Matrix, Sensory Modality and Rehabilitation Technique, the Sensory Stimulation Assessment Measure (Opara et al., 2014), and Western Neurosensory Stimulation Profile (Horn et al., 1993), which evaluate the overall outcome and functional status of patients post-injury, providing valuable insights into the long-term effects and recovery trajectory.

However, it is important to note that subjective interpretation, consciousness fluctuations, and the inability of patients to respond during behavioral assessments can lead to misclassification errors, which occur in approximately 37% to 40% of cases (Jain and Ramakrishnan, 2020). To minimize these errors, a combination of diverse neuroimaging and electrophysiological techniques can be utilized to enhance the accuracy of assessing levels of consciousness (Jain and Ramakrishnan, 2020). Electroencephalography (EEG) signals have emerged as a critical input in the clinical assessment of DOC patients (Edlow et al., 2021). Being a non-invasive neuroimaging technique, EEG provides insights into the underlying brain activity and connectivity, that help understand the neurological mechanisms governing consciousness. It plays a multifaceted role in the clinical evaluation of DOC patients, offering diagnostic clarity and aiding in the prediction of potential recovery (Subha et al., 2010). The historical evolution of EEG-based visual analysis methods for categorizing patients with consciousness disorders dates back to 1965, marked by subsequent refinements in 1988 and 1997 (Young et al., 1997; Bai et al., 2021b). In 2012, EEG terminology was standardized to improve consistency, which was complemented by additional scoring scales in 2014 and 2016 (Estraneo et al., 2016), fostering a systematic EEG interpretation approach (Hirsch et al., 2021). However, challenges still persist in visual analysis methods of consciousness disorders, such as subjectivity, limited predictive accuracy, and a diverse patient population. Overcoming these challenges requires a comprehensive strategy, incorporating machine learning for objectivity, advanced EEG measures, tailored classifications, standardized protocols, continuous monitoring, and biomarker integration. These challenges can be tackled by integrating diverse signal processing methods to analyze brain signals across different domains namely time, frequency, and time-frequency domains.

Among various EEG paradigms to study DOC patients, resting-state EEG-based analysis has gained attention as it does not require active task performance or external stimulation, making it an ideal tool for patients who are unable to follow commands or respond to stimuli due to their compromised consciousness (Chen et al., 2022).

Spectral power analysis, a component of frequency domain analysis, employs techniques such as the Fourier Transform to convert time-domain signals into their frequency representation. This facilitates the identification of variations in different frequency bands such as alpha, delta, and theta bands, distinguishing between patients in unresponsive wakefulness syndrome (UWS) and minimally conscious state (MCS) (Bai et al., 2021b). Studies reveal increased powers of delta and theta bands in MCS compared to severe neurocognitive disorder patients, while UWS patients exhibit decreased alpha but increased delta power compared to MCS patients, along with variations in theta power (Bai et al., 2021b). Limited exploration is reported for beta and gamma band frequencies, and normalized powers show distinctions between consciousness states, particularly in delta, theta, and alpha bands (Corchs et al., 2019).

EEG microstates, representing stable scalp potential fields, correlate with altered states of consciousness and unawareness (Stefan et al., 2018). Reduced microstate types and diminished diversity in alpha-rhythmic microstates are associated with altered consciousness states. The percentage of time spent in microstate D in the alpha frequency band is a key discriminator between UWS and MCS patients (Bai et al., 2021a).

Functional connectivity assesses the integration of brain networks and encounters challenges in DOC patients. Measures like coherence, imaginary coherence (IC) (Stefan et al., 2018), phase lag index (PLI) (Sitt et al., 2014), and directed transfer function (Höller et al., 2014) have been employed to analyze EEG resting-state connectivity. Among patients with disorders of consciousness, MCS patients exhibit heightened connectivity in alpha and beta bands relative to UWS patients. Coherence analysis struggles to differentiate between MCS and UWS patients, while cross-approximate entropy reveals suppressed and increased interconnections in UWS and MCS patients, respectively (Bai et al., 2021b). Outgoing Granger causality is wider than incoming values for UWS, MCS, EMCS, and controls, and dissymmetry is observed in outgoing and incoming information in UWS patients (Lee et al., 2013). Transfer entropy faces challenges in distinguishing groups, with weighted symbolic mutual information offering independence from etiology. Biomarker analysis successfully distinguishes UWS from MCS and controls. Dynamic functional connectivity reveals significant temporal differences between MCS and UWS, which correlates with CRS-R. In specific connectivity measure comparisons, Höller et al. (2014) find partial coherence to yield optimal results, with directed transfer function and generalized partial directed coherence also effectively differentiating UWS from MCS patients. Lehembre et al. (2012) emphasize the equal suitability of IC and PLI in distinguishing UWS from MCS in low-density EEG, overcoming coherence limitations related to volume conduction problems. Stefan et al. (2018) conduct a comprehensive analysis combining various indices, determining the percentage of time spent in alpha microstates as optimal for distinguishing UWS from MCS patients. In contrast, the clustering coefficient, focusing on beta coherence, exhibits a higher predictive value for outcomes.

Graph theory involves nodes and connections to provide insights into altered functional connectivity in DOC patients (Chennu et al., 2017). In the alpha band, reduced local/global efficiency and fewer hubs are observed, with distinct modules. Differences in network metrics indicate altered connectivity and discriminate between UWS and MCS patients. DOC patients exhibit impaired network integration, and their consciousness level correlates with altered network dynamics. Naro et al. (2021) studied functional connectivity in resting-state EEG among 17 UWS patients and 15 MCS patients based on multiplex and multilayer network analyses of frequency-specific and area-specific networks. The findings revealed the degradation and heterogeneity of functional networks, particularly in the fronto-parietal region, serving as a discriminant between patients with MCS and UWS. However, these insights were not discernible when examining each frequency-specific network.

Non-linear analysis of transformed EEG signals reveals several measures that effectively discriminate between unconscious and conscious patients while also correlating with different levels of consciousness (Bai et al., 2021a). The Kolmogorov Chaitin Complexity (KCC) stands out for successfully differentiating between UWS and MCS patients, particularly in the parietal region (Bai et al., 2021a). Diverse metrics, including Lempel-Ziv complexity, approximate entropy, and cross-entropy, consistently demonstrate their effectiveness in distinguishing between UWS and MCS (Duszyk-Bogorodzka et al., 2022). Permutation entropy-based measures, especially in the theta range, prove effective in distinguishing UWS patients (Engemann et al., 2018). Additionally, MCS patients exhibit a higher mean spectral entropy than UWS patients, offering further insights into different consciousness states. Higher entropy values, indicative of a less regular resting EEG, are associated with proximity to an awake state, while lower values correlate with unconscious states (Engemann et al., 2018).

Time-frequency analysis methods, such as wavelet transforms, empirical mode decomposition (EMD) (Huang et al., 1998), and variational mode decomposition (VMD) (Dragomiretskiy and Zosso, 2013), play a pivotal role in the processing and interpretation of electroencephalogram signals. These techniques address the inherent non-stationarity and complexity of EEG data, offering a dynamic perspective on the brain's temporal and spectral characteristics. In general, they decompose a given signal into a collection of individual components known as modes and each mode represents a distinct oscillatory behavior or pattern within the signal. The wavelet transform (Daubechies, 1992) allows for time-frequency analysis of the EEG signal by decomposing it into different scales or frequency bands (Subasi et al., 2021). Advanced wavelet decomposition techniques, including the flexible analytic wavelet transforms, adaptive flexible analytic wavelet transform (Khare and Acharya, 2023), tunable Quality Factor (Puri et al., 2022), adaptive tunable Q wavelet transform, and rational dilation wavelet transform (Taran et al., 2020), were employed to investigate EEG signals in diverse clinical and non-clinical contexts. These techniques were applied in studies involving patients with conditions such as hypertension (El-Dahshan et al., 2024), attention-deficit/hyperactivity disorder (Khare et al., 2023), schizophrenia (Khare and Bajaj, 2021), and Alzheimer's (Puri et al., 2022). Apart from this, these advanced wavelet decomposition methods found utility in the domain of emotion recognition (Khare et al., 2024), motor imagery (Taran et al., 2020), and automatic selection of tuning parameters for decomposing EEG signals (Khare and Acharya, 2023).

Empirical mode decomposition is a data-driven and adaptive technique that partitions the EEG signal into intrinsic mode functions (IMFs) (Sweeney-Reed et al., 2018). IMFs represent the different oscillatory components present in the signal, capturing both stationary and non-stationary features (Carvalho et al., 2020). By examining the amplitude, frequency, and energy distribution of the IMFs, clinicians can identify abnormalities or distinctive patterns that aid in diagnosis and prognosis. EMD has been successfully applied to diverse neurological conditions, including epilepsy, sleep disorders, and neurodegenerative diseases (Ren et al., 2016), and other applications like emotion recognition (Salankar et al., 2021), and brain-computer interface (Hsu et al., 2020; Dash et al., 2022). Numerous expansions and variations of EMD method have been suggested as a means to enhance its applicability in EEG signal processing. One notable extension is the ensemble empirical mode decomposition (EEMD) (Sweeney-Reed et al., 2018). EEMD generates multiple realizations of the EEG signal by adding white noise and applies EMD to each realization. The resulting IMFs are then averaged to obtain a more robust decomposition. Complementary ensemble empirical mode decomposition (Yeh et al., 2010) decomposes the signal into IMF components using both EMD and complementary EMD and combines the corresponding IMFs to improve mode separation. These extensions of the EMD method provide valuable alternatives for EEG analysis, overcoming some of the limitations associated with traditional EMD and enabling more accurate decomposition results. Furthermore, complete ensemble empirical mode decomposition with adaptive noise (Jia et al., 2017) extends EMD by utilizing a noise-assisted approach similar to EEMD and adaptively adjusting the added noise to improve decomposition accuracy. These extensions of the EMD method offer valuable tools for analyzing EEG signals, facilitating the extraction of relevant temporal and spectral information from complex and non-stationary brain activity (Jia et al., 2017).

Variational mode decomposition (Dragomiretskiy and Zosso, 2013) introduces a variational principle to enhance mode separation and has been developed recently. VMD incorporates a regularization term that promotes smoothness in the decomposed IMFs, leading to improved signal decomposition. This eliminates the drawbacks of EMD like mode mixing, inability to cope with noise, and recursive sifting in most methods that do not allow backward error correction (Carvalho et al., 2020). VMD has been applied for processing and interpretation of EEG for various applications like epilepsy (Qin et al., 2023), seizure detection (Mathew et al., 2023), emotion detection (Khare and Bajaj, 2020b; Liu et al., 2023), ADHD (Khare et al., 2022), schizophrenia (Siuly et al., 2020), Alzheimer's disease diagnosis (Aslan, 2023), drowsiness detection (Khare and Bajaj, 2020a), and sleep stage classification (Che et al., 2023). In addition to processing EEG signals, the VMD technique has been utilized in the analysis of speech (Upadhyay and Pachori, 2015) and the detection of glaucoma using fundus images (Maheshwari et al., 2017).

The examination of the literature indicates a gap in the exploration of time-frequency-based analysis of EEG signals for diagnosing and classifying patients with disorders of consciousness. While variational mode decomposition has seen widespread applications in various clinical and non-clinical domains, its utilization in the context of DOC remains limited or non-existent. VMD, particularly in the realm of EEG signals, has been predominantly employed in studies related to epilepsy and Alzheimer's disease.

This study aims to address this gap through exploratory analysis assessing the adaptive decomposition capabilities of variational mode decomposition when integrated with machine-learning models to classify patients with impaired consciousness. The research also delves into the effectiveness of alternative feature extraction and classification techniques for different categories of disorders of consciousness. Additionally, it compares the performance of VMD-derived features with those of the conventional approaches based on frequency bands and raw EEG. The overarching objective is to provide a supportive tool for behavioral assessment methods and enhance the understanding of the neurological conditions affecting individuals with disorders of consciousness.

## 2 Methodology and data collection

### 2.1 Clinical EEG data acquisition protocol

The EEG recording was conducted at NIMHANS using the EBNeuro Galileo NT Line 3.90 device equipped with EEG.NET software, specifically the EEG Glant version. The procedure was carried out under the supervision of an EEG technician. The equipment utilized was a standard 32-channel system, inclusive of the DC channel MK, with additional ECG and EKG channels. The electrode placement during recording followed the international 10/20 system at a sampling rate of 256 Hz, with reference electrodes placed on the left and right ear lobes (A1, A2), maintaining an impedance below 5K Ohm. The steps followed for EEG recording are as per the work (Raveendran et al., 2023).

### 2.2 Patients

Forty-five participants, aged 18 years and above, meeting the inclusion/exclusion criteria for disorders of consciousness, were enrolled in the study. Within the patient group, 15 individuals were in a coma state, while 15 each were in MCS and UWS. The average age of the participants was 42.95 years, comprising 24 males and 21 females. During the recording session, subjects were instructed to assume a dorsal decubitus position and keep their eyes closed. Both the healthy and patient cohorts underwent a recording duration of 30 min. The patient recruitment process involved a team of experts, including neurologists and neuro-anesthetists. The focus was on inpatients admitted to Neuroscience services, specifically neurology wards and intensive care units, exhibiting consciousness disorders due to various factors, both traumatic and non-traumatic. To classify the recruited patients, their CRS-R and Glasgow coma scale scores were calculated, aiding in determining their placement into different categories of disorders of consciousness. The following inclusion and exclusion criteria were followed during the patient recruitment process:

#### 2.2.1 Inclusion criteria

Adult patients, aged 18 years and above, who have been diagnosed to be in coma, UWS, or MCS condition, as per the guidelines provided by the American Academy of Neurology were recruited for the study. The research also encompassed patients with DOC arising from various underlying brain diseases, including stroke, encephalitis, status epilepticus, and anoxia, as well as both traumatic and non-traumatic causes. Patients or their caregivers were required to provide informed consent to participate in the study. The study included acute and chronic cases with varying durations post-brain injury, where the acute cases lasted for more than 7 days.

#### 2.2.2 Exclusion criteria

The study excluded patients who met any of the following criteria: those with medically unstable conditions that make EEG recording impractical, individuals with progressive neurodegenerative conditions, those with reversible and acute causes of DOC with less than 7 days from the start of brain injury, non-cooperative patients, and patients without informed consent from the caregiver. Furthermore, individuals under the influence of drugs were excluded from the study cohort. The recruitment criteria further specified the inclusion of only those patients who maintained stability for a consecutive three-day period with consistent CRS profiles, and a commitment to a follow-up period of six months to ensure a more reliable and comprehensive dataset. The detailed demographics of the recruited patients are listed in Table 1.

**Table 1:** Demographics of the 45 DOC patients included in the study.

**Etiology**	**Age**	**Gender**		**DOC category**		
		**Male**	**Female**	**Coma**	**UWS**	**MCS**
Acute disseminated encephalomyelitis	54	0	1	0	1	0
Aneurysmal intracranial hemorrhage	55	1	0	0	0	1
Autoimmune-CNS lupus	25	1	1	1	1	0
Bacterial meningitis	20	1	0	1	0	0
Cerebral venous thrombosis	49	1	2	0	0	3
Demyelination	31	1	0	0	0	1
Encephalopathy	46	5	3	1	5	2
Frontoparietal bleed	91	1	0	1	0	0
hypoxia	48	0	1	0	0	1
Hypoxic ischemic encephalopathy	27	1	2	1	2	0
Intracranial hemorrhage	54	2	1	0	1	2
Mass lesion	33	0	1	0	1	0
Meningioma	24	1	0	1	0	0
Meningoencephalitis	37	1	2	1	1	1
Metabolic/toxic encephalopathy	34	3	1	2	2	0
Stroke	69	2	2	2	0	2
Sub arachnoid hemorrhage	70	0	1	0	1	0
Traumatic brain injury	40	1	0	1	0	0
Tubercular meningitis	46	1	0	0	0	1
Tubercular meningitis with hydrocephalus	33	0	3	2	0	1
Vestibular schwannoma	57	1	0	1	0	0

### 2.3 EEG data pre-processing

The initial phase of signal pre-processing after data acquisition involved the visual analysis of raw EEG data by skilled EEG technicians at NIMHANS. Their primary objective was to identify and flag artifactual epochs and problematic channels within each signal. Epochs that exhibited significant levels of artifacts were excluded from subsequent pre-processing procedures. The average referencing step of preprocessing was followed by a bandpass filter with a low cut-off at 0.1 Hz and a high cut-off at 45 Hz to eliminate the powerline artifact at 50 Hz. Eye blink artifacts were mitigated using independent component analysis. Since some channels were unavailable in the EEG records of some coma patients; to maintain uniformity in the data for further analysis, the number of channels was reduced to 17 (F3, Fz, F4, F7, F8, Cz, C3, C4, T3, T4, T5, T6, P3, Pz, P4, O1, and O2). The basic analysis of the resting state EEG of DOC patients for the classification is performed as reported in the previous work (Raveendran et al., 2023). The pre-processing steps were carried out using Python on the Anaconda Jupyter online platform.

## 3 Proposed approach

**Figure 1:**
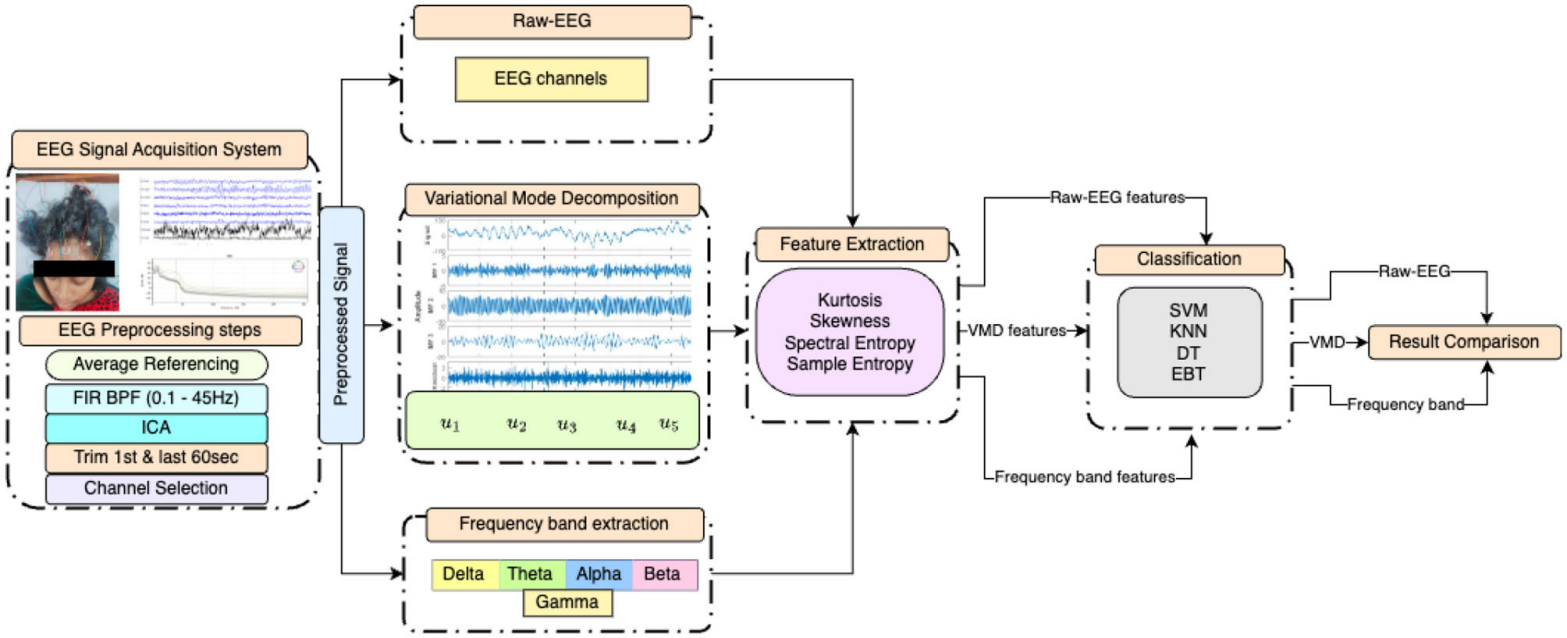
Block diagram of the experiments conducted, illustrating the preprocessing steps, statistical feature extraction from EEG after three distinct initial processings, and the four classifiers employed.

This paper proposes an exploratory study on how VMD can be used as an analytical tool for the resting state EEG of DOC patients. The performance of various classifiers is measured based on features extracted from VMD modes. This performance is then compared with those of the features extracted from different frequency bands of EEG as well as raw EEG of the 17 channels. The block diagram presented in Figure 1 depicts the proposed approach and the different experiments conducted in graphical form.

### 3.1 Variational mode decomposition

Variational mode decomposition is a signal processing algorithm designed to decompose a given signal into a finite number of oscillatory modes, each characterized by its central frequency, amplitude, and phase. These modes are extracted through an iterative optimization process that seeks to minimize the cross-term interference among the modes while preserving their characteristics (Dragomiretskiy and Zosso, 2013). VMD can be used to manage signal variability and artifacts by separating the signal components that correspond to different sources of information or noise. The advantages of VMD over other methods in managing signal variability and artifacts are:

- VMD adapts to the signal and does not need any prior knowledge of the signal or any a priori defined basis functions.
- VMD avoids the problem of modal mixing that occurs when a single mode contains signals from different sources or scales. This improves the accuracy and robustness of the signal decomposition and artifact removal.
- VMD can be combined with other methods, such as blind source separation, to further enhance the performance of artifact removal.

At the same time, VMD also has certain limitations:

- It is dependent on two parameters, namely the number of modes and the penalty factor, which affect the quality of the decomposition and artifact removal. These parameters need to be precisely chosen or optimized for each scenario and application.
- VMD may not be able to handle non-stationary signals with time-varying frequency or amplitude. This reduces the effectiveness of VMD in handling signal variability and artifacts in some cases.

The variational mode decomposition algorithm can be summarized as follows:

#### 3.1.1 Signal decomposition

Given a signal *x*(*t*), the VMD algorithm aims to find K oscillatory modes *u*_*k*_(*t*) and corresponding central frequencies ω_*k*_ such that:


(1) $$\begin{array}{l} x\left(t\right)=\overset{K}{\underset{k=1}{\sum }}u_{k}\left(t\right) \end{array}$$

where *k* indexes the modes, and *K* is the desired number of modes.

#### 3.1.2 Initialization

To start the iterative process, the algorithm requires an initial estimate of the modes. This can be achieved using any method that provides a reasonable initial approximation.

#### 3.1.3 Optimization

The core of VMD lies in the optimization step, where it refines the estimates of the modes and central frequencies. This step involves solving an optimization problem to minimize a cost function that encourages both spatial and spectral sparsity while maintaining the fidelity of the signal reconstruction. The cost function *J* is defined as:


$$\begin{array}{l} J=\sum_{k=1}^{K}\|\partial_{t}\left[\left(\delta (t)+\frac{j}{\pi t}\right)*u_{k}(t)\right]{e^{-j\omega_{k}t}\|}_{2}^{2} \end{array}$$

The minimization problem min(*u*_*k*_, ω_*k*_){*J*} subject to Equation 1 is solved by finding the saddle point of augmented Lagrangian L using alternate direction method of multipliers. This is defined as,


(2) $$\begin{array}{l} ℒ(u_{k},\omega_{k},\lambda )=\alpha \sum_{k=1}^{K}\|\partial_{t}\left[\left(\delta (t)+\frac{j}{\pi t}\right)*u_{k}(t)\right]\;{e^{-j\omega_{k}t}\|}_{2}^{2}\\ \;+\|x(t)-\sum_{k=1}^{K}{u_{k}(t)\|}_{2}^{2}+\langle \lambda (t),x(t)-\sum_{k=1}^{K}u_{k}(t)\rangle \end{array}$$

where;

- *x*(*t*) is the original signal.
- *u*_*k*_ represents the k-th mode.
- ||·||_2_ denotes the L2 norm.
* denotes the convolution operator.
- λ and α are regularization parameters controlling the sparsity and cross-term interference terms, respectively.

#### 3.1.4 Mode update

After optimizing the cost function, the algorithm updates the modes *u*_*k*_ and central frequencies ω_*k*_ to better fit the data. This process continues iteratively until one of the criteria of relative tolerance ε_*r*_ or absolute tolerance ε_*a*_ is met;


(3) $$\begin{array}{l} \underset{k}{\sum }||u_{k}^{n+1}\left(t\right)-u_{k}^{n}\left(t\right)|{|}_{2}^{2}/||u_{k}^{n}\left(t\right)|{|}_{2}^{2}<\varepsilon_{r} \end{array}$$

(4) $$\begin{array}{l} \underset{k}{\sum }||u_{k}^{n+1}\left(t\right)-u_{k}^{n}\left(t\right)|{|}_{2}^{2}<\varepsilon_{a} \end{array}$$

#### 3.1.5 Reconstruction

Once the iterations converge, the final decomposed modes and their central frequencies are used to reconstruct the original signal. The reconstructed signal $\hat{x}\left(t\right)$ is obtained by summing up all the modes:


$$\hat{x}\left(t\right)=\overset{K}{\underset{k=1}{\sum }}u_{k}\left(t\right)$$

### 3.2 EEG frequency band based approach

The EEG signal in general can be said to have five different frequency bands. One of the primary frequency bands examined in resting-state EEG analysis of DOC patients is the delta band (0.5–4 Hz). Delta oscillations are typically associated with deep sleep and are characterized by high-amplitude, slow-frequency waves. In DOC patients, increased delta power during resting-state EEG may suggest a profound impairment of consciousness, resembling the patterns seen during deep non-REM sleep. Theta band activity (4–8 Hz) is another crucial aspect of resting-state EEG analysis in DOC patients. Theta waves are associated with memory processes, attention, and cognitive engagement. Alpha band (8–13 Hz) activity plays a pivotal role in resting-state EEG analysis, as it is linked to the brain's idling state during relaxation and wakefulness with closed eyes. The presence of alpha activity, particularly over posterior regions of the brain, suggests a degree of preserved cortical functioning. The beta band (13–30 Hz) in resting-state EEG analysis is associated with active thinking, problem-solving, and alertness. The presence of beta activity in DOC patients, especially over frontal regions, may suggest residual cognitive processing. The gamma band (30–40 Hz) oscillations are associated with information binding, sensory integration, and conscious perception. The presence of gamma activity, particularly over parietal and frontal regions, may indicate the potential for higher-level cognitive processing and awareness (Ballanti et al., 2022).

The frequency bands were extracted using the bandpass filters with a specific range of bands.

## 4 Feature extraction

Four statistical features, namely spectral entropy (*E*_*S*_), sample entropy (*E*_*S*_*am*), skewness (*Sk*), and kurtosis (*K*) were considered for the study. Sample entropy and spectral entropy are two key concepts in complexity analysis and signal processing. The features are extracted from each mode for the VMD-based approach, from each frequency band for the frequency band-based approach, and from raw EEG of each channel to understand the feature variation in each group of patients.

### 4.1 Spectral entropy

Spectral entropy (*E*_*S*_), a normalized adaptation of Shannon's entropy, utilizes the amplitude components of the power spectrum derived from time series data to evaluate entropy (Gibson, 1994). It measures the complexity or irregularity of the frequency content within a specific frequency band of the EEG signal. To calculate *E*_*S*_, one multiplies the normalized power within each frequency component by the logarithm of that power and then negates the product. The formula for spectral entropy as per (Schmierer et al., 2022) is represented as:


$$E_{S}=-\sum (P(f)\cdot \log(P(f))$$

Higher spectral entropy values in certain frequency bands indicate a more conscious state, while lower values suggest a diminished level of consciousness.

### 4.2 Sample entropy

Sample entropy (*E*_*Sam*_) is defined as a metric for assessing the intricacy of time series data. This metric is a modified version of approximate entropy and offers several advantages over its predecessor. Unlike approximate entropy, sample entropy is not contingent on the length of the time series, is devoid of self-counting, and boasts straightforward implementation. Mathematically, sample entropy is expressed as the negative natural logarithm of the conditional probability that two sequences, each similar for m data points, remain similar at the next point (m+1). Notably, self-matches are not excluded in this computation. Furthermore, sample entropy is known for preserving a sense of relative consistency, and its key parameters, m (representing the run length of the vector under analysis), r (the tolerance window), and N (the total number of data points), play a pivotal role in determining its statistical properties.


$$\begin{array}{l} \;E_{Sam}(m,r,N)=-log\frac{A}{B}\\ where\;A=C(m+1,r),\;B=C(m,r) \end{array}$$

### 4.3 Skewness

Skewness (*S*_*k*_) is a higher-order moment, which measures the asymmetry of a probability distribution, indicating whether the distribution is skewed to the left or right. In the context of EEG signals, skewness reflects the underlying asymmetry or imbalance in the neural activity within different frequency components (Zhao et al., 2023). If the input EEG data is distributed more towards the left of the mean point, it has a positive skewness value. If it is distributed more to the right side of the mean, the skewness value is negative. Skewness is defined as,


(5) $$\begin{array}{l} S_{k}=\frac{E\left[{\left(x\left(n\right)-\mu \right)}^{3}\right]}{\sigma^{3}} \end{array}$$

where E is the expected value and σ is the standard deviation. High skewness values in certain frequency bands indicate an imbalance or abnormality in neural activity, suggesting a diminished level of consciousness. Conversely, lower skewness values indicate a more symmetrical distribution of activity, associated with a more conscious state (as shown in Equation 5).

### 4.4 Kurtosis

Kurtosis (*K*) measures the “peakedness” or “tailedness” of a probability distribution, providing insights into the shape and distribution of the underlying neural activity within different frequency components (Zhao et al., 2023). In the context of EEG signals, kurtosis reflects the deviation of the distribution from a Gaussian distribution. High kurtosis values indicate a more peaked or heavy-tailed distribution, suggesting the presence of transient changes in neural activity. On the other hand, low kurtosis values indicate a flatter distribution, implying a more stable and consistent pattern of neural activity. Kurtosis within each VMD mode is computed as,


(6) $$\begin{array}{l} K=\frac{E\left[{\left(x\left(n\right)-\mu \right)}^{4}\right]}{{\left[E\left[{\left(x\left(n\right)-\mu \right)}^{2}\right]\right]}^{2}} \end{array}$$

Higher kurtosis values in certain frequency bands may indicate the presence of irregular or non-stationary activity, which could be associated with a diminished level of consciousness. Lower kurtosis values, indicating a more stable and consistent distribution (as shown in Equation 6), may be indicative of a more conscious state (Khoshnevis and Sankar, 2019).

## 5 Frequency band analysis vs. VMD modes

Analyzing resting-state EEG signals in patients with disorders of consciousness using traditional EEG frequency bands and variational mode decomposition modes reveals distinct perspectives on brain activity. It can offer complementary insights into the neural dynamics underlying these complex conditions. Traditional EEG frequency bands have long been used to characterize the spectral content of EEG signals. These bands can provide valuable information about the patient's level of consciousness and cognitive functioning. On the other hand, variational mode decomposition is a novel signal-processing technique that is mainly used for non-stationary signals like EEG. Comparing the two approaches, traditional EEG frequency bands provide a well-established framework for categorizing brain activity based on frequency ranges. However, they oversimplify the complexity of resting-state EEG signals in DOC patients, as they assume predefined frequency ranges that do not always capture the full spectrum of neural dynamics. In contrast, VMD modes offer a more flexible and data-driven approach, allowing researchers to uncover hidden components that traditional frequency bands might miss.

## 6 Choice of machine learning models

For classifying the DOC cohort based on VMD mode-based, frequency band-based, and raw EEG-based features, two types of classifications are carried out. In one, binary classification is performed between the UWS and MCS categories. In the other, multi-class classification is carried out among the coma, UWS, and MCS categories. For binary classification, the dataset included 30 patients, while for multiclass classification, the dataset comprised 45 patients. The choice of specific models for a machine learning task needs to take into consideration the nature of the data and the study's objectives. Since obtaining EEG data on DOC patients is a difficult task, there are no publicly available databases with a sufficiently high number of patients to be able to use sophisticated classifiers such as end-to-end deep learning-based models. So, we take resort to conventional machine learning models. The classifier model we are looking for needs to handle only two or three classes.

We intended to employ multiple classifiers (Duda et al., 2021) in order to compare their performances on the different approaches and identify the best of them for the task at hand. Decision trees (DT) are appropriate for classification problems where the training data may contain errors or noise, and thus we chose them as one possible classifier. Whether one needs to perform binary or multi-class classification, the K-nearest neighbor algorithm (KNN) works well. Thus KNN was selected as another classifier. Support vector machine (SVM) is a powerful machine learning algorithm that has been used for a variety of classification tasks. SVMs are efficient and adaptable since they can handle high-dimensional data and nonlinear relationships. SVM is very effective since it finds the maximum separating hyperplane between the different classes in the feature space. So, SVM is selected as the third classifier. An ensemble bagged tree (EBT) classifier combines the predictions of many decision trees that are trained on random subsets of the data and features, which reduces the effects of overfitting and improves generalization. Thus, we included EBT also as a classifier. Thus, four different classifiers are employed and the performance of the classifiers is assessed using various metrics, namely accuracy, precision, recall, and F1-score.

## 7 Results

The research studies and compares the performances of features obtained from five VMD modes, EEG frequency bands (delta, theta, alpha, beta, and gamma), and the 17 channels chosen for analysis. The extracted features consist of spectral entropy, sample entropy, kurtosis, and skewness. Python was utilized for data analysis and classification. Before extracting VMD modes and frequency bands, the variations in features between groups were scrutinized for each channel. Subsequently, the classification of disorders of consciousness groups, covering both binary and multi-class scenarios, was carried out. A 10-fold cross-validation was performed to prevent overfitting.

### 7.1 Choice of machine learning models

KNN, SVM with linear kernel, DT, and EBT classifiers were considered for both binary and multi-class classifications.

### 7.2 Approach 1: features derived from the raw EEG

**Figure 2:**
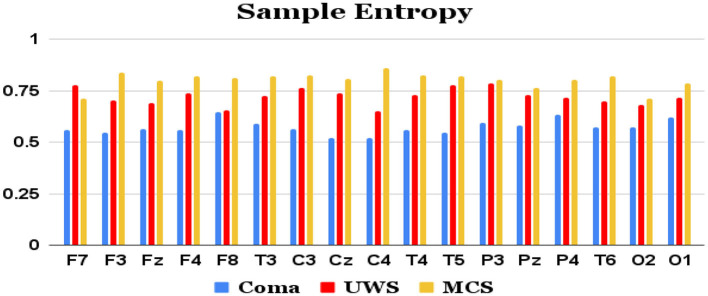
Mean sample entropy values (y-axis) of each group of patients computed from each of the 17 raw EEG channels (x-axis).

**Figure 3:**
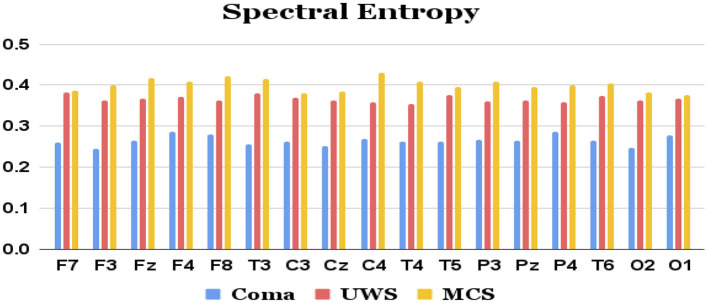
Mean spectral entropy values (y-axis) of each group of patients extracted from each of the 17 channels (x-axis) of raw EEG.

**Figure 4:**
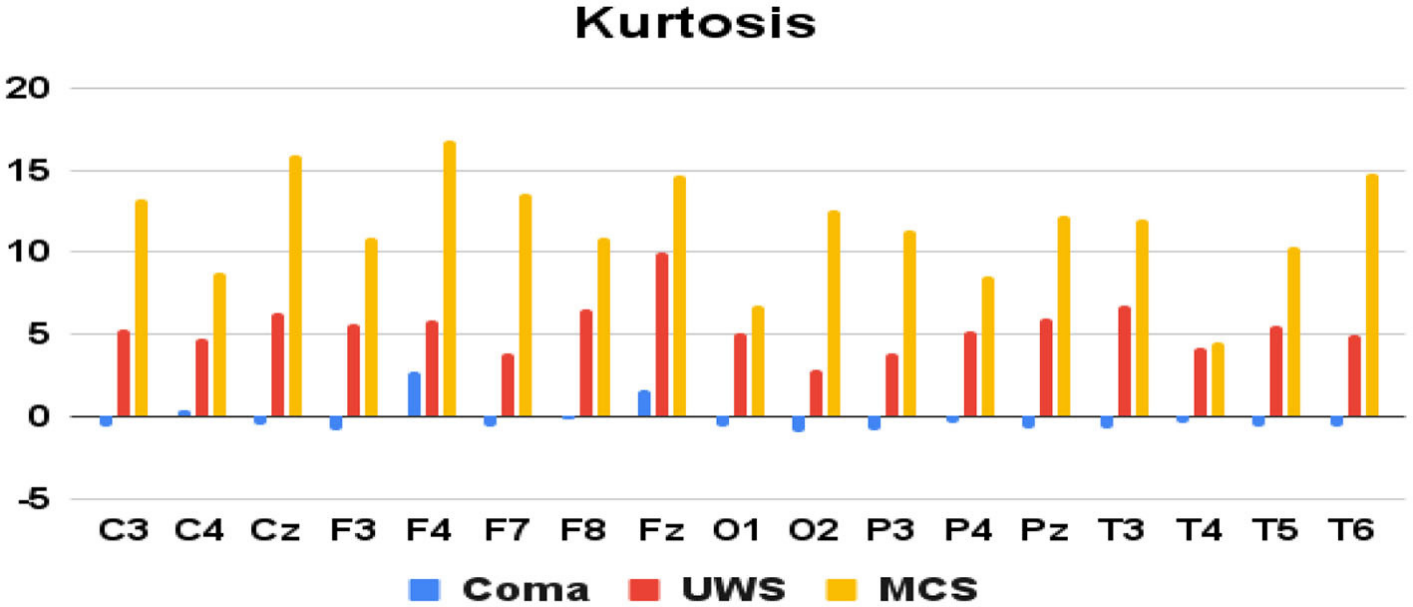
Mean kurtosis values (y-axis) of each group of patients derived from each of the 17 raw EEG channels (x-axis).

**Figure 5:**
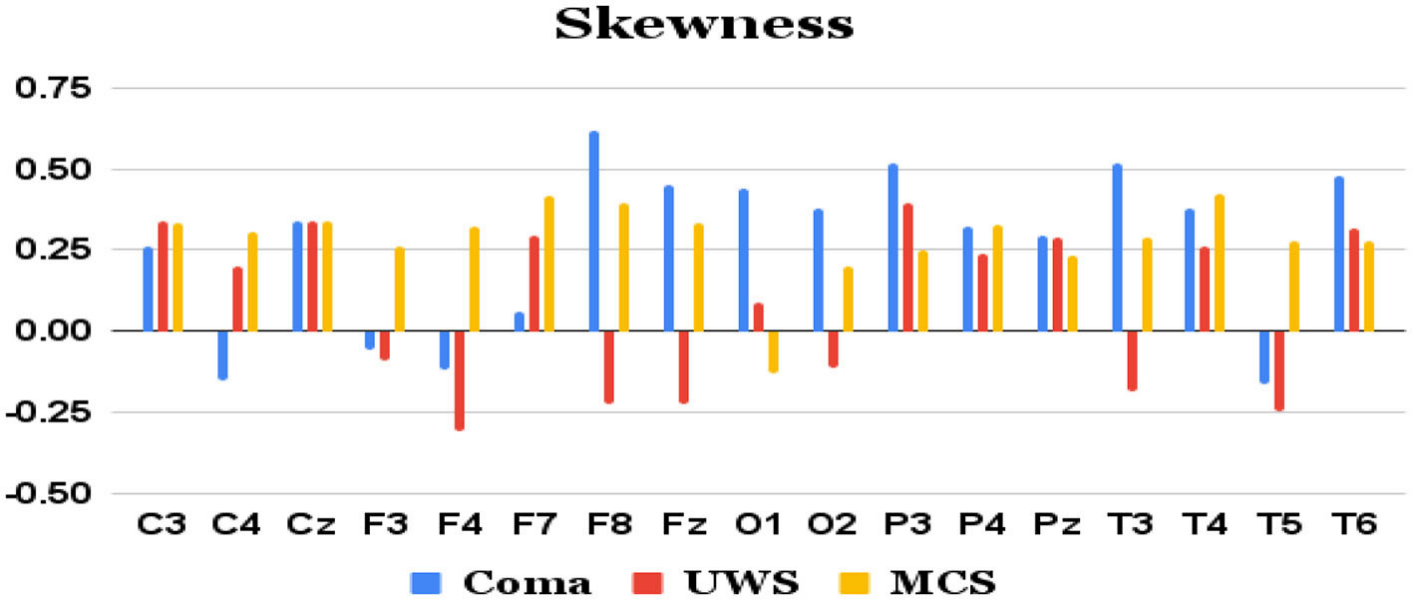
Mean skewness values (y-axis) of each group of patients derived from each of the 17 channels (x-axis) of raw EEG.

Features extracted from the preprocessed EEG of all the 17 channels (a total of 17 × 4 = 68 features) are analyzed to observe the variations of each feature between the 3 patient groups. It was observed that the MCS patients had more complex EEG signals indicating better awareness and wakefulness than the coma and UWS groups. Figures 2, 3 illustrate the variation in the mean values of the sample entropy and spectral entropy features among the three patient groups for each channel. Similarly, the variations in the mean values of kurtosis and skewness are displayed in Figures 4, 5, respectively. The features extracted from each channel were then considered as input to the various classifiers and both binary and multi-class classification were performed. Table 2 shows the binary classification outcomes between UWS and MCS, leveraging features extracted from the chosen 17 EEG channels. In this analysis, Li.SVM emerges as a standout, achieving an accuracy of 73.3%. It also demonstrates higher precision, recall, and F1 scores, attesting to its better performance in identifying UWS and MCS states. Table 3 showcases the outcomes of the multi-class classification endeavor between coma, UWS, and MCS patients. Ensemble of bagged trees and KNN perform better than the other two classifiers but achieve accuracy values of 45.5% and 46% only.

**Table 2:** Binary (UWS vs. MCS) classification results (in %) using 10-fold cross-validation with all four features directly extracted from the 17 channels of the raw EEG.

**Classifier**	**Accuracy**	**Precision**	**Recall**	**F1 score**
KNN	56.7	45	55	49.5
Li.SVM	73.3	65.8	72.5	69
DT	53.3	47.5	52.5	49.9
EBT	60	55.8	62.5	59

Number of patients: 30.

**Table 3:** Multi-class (Coma vs. UWS vs. MCS) classification results (in %) using 10-fold cross-validation with all the four features extracted from the 17 channels of the raw EEG.

**Classifier**	**Accuracy**	**Precision**	**Recall**	**F1 score**
KNN	46	37.2	48.3	42
Li.SVM	35.5	28.3	40	33.1
DT	40	31.8	41.7	36.1
EBT	45.5	40.7	48.3	44.2

Number of patients: 45. The accuracy achieved with every classifier is below par.

### 7.3 Approach 2: features derived from the frequency bands

The five distinct frequency bands of the EEG signal were derived from the preprocessed signals employing a zero-phase, non-causal FIR bandpass filter. The filter utilized a windowed time-domain design method (firwin) with lower and upper cut-off frequencies corresponding to various EEG bands. Specifically, a Hamming window with a 0.0194 passband ripple and 53 dB stopband attenuation was employed. Subsequently, the same four features were extracted from each of these frequency bands (a total of 17 channels × 5 bands × 4 = 340 features) and utilized as inputs to the classifiers.

Table 4 shows the performance of the frequency band derived features in the binary classification between UWS and MCS. For the binary classification, KNN achieved a low accuracy of 36.7%. In contrast, Li.SVM demonstrated higher accuracy, reaching 66.7%, suggesting a more accurate classification than KNN. Decision tree and EBT achieved lower performance with accuracies of 30% and 36.7%, respectively. KNN exhibited precision, recall, and F1 scores of 35.8%, 42.5%, and 38.5%, respectively. These metrics show KNN's poor ability in making positive predictions, identify actual positives, and achieve a balanced measure of precision and recall. Li.SVM demonstrated somewhat better performance, with precision, recall, and F1 scores of 58.3%, 75%, and 65.6%, respectively. DT demonstrated poor performance across precision, recall, and F1 scores, with values of 26.7%, 30%, and 28.3%, respectively. EBT showcased precision, recall, and F1 scores of 25.8%, 37.5%, and 30.6%.

**Table 4:** Binary (UWS vs. MCS) classification results (in %) using 10-fold cross-validation with the entire set of frequency band-based features.

**Classifier**	**Accuracy**	**Precision**	**Recall**	**F1 score**
KNN	36.7	35.8	42.5	38.5
Li.SVM	66.7	58.3	75	65.6
DT	30	26.7	30	28.3
EBT	36.7	25.8	37.5	30.6

Number of patients: 30. The performance is inadequate with all the classifiers.

Table 5 lists the performance of the four classifiers in the multi-class classification task between coma, UWS, and MCS patients. Support vector machine with linear kernel performed better in distinguishing among coma, UWS, and MCS, than the other three classifiers, but achieved a poor accuracy of 45% only.

**Table 5:** Multi-class (Coma vs. UWS vs. MCS) classification results (in %) using 10-fold cross-validation for the entire set of frequency band-derived features.

**Classifier**	**Accuracy**	**Precision**	**Recall**	**F1 score**
KNN	34.5	32.5	35	33.7
Li.SVM	45	36.1	41.7	38.7
DT	43.5	36.1	43.3	39.4
EBT	42.5	34.2	45	38.9

Number of patients: 45. The performance is unsatisfactory with all the classifiers.

### 7.4 Approach 3: features derived from VMD modes

In this approach, the same four features were extracted from each of 5 VMD modes (a total of 17 channels × 5 IMFs × 4 = 340 features). The penalty factor α was considered 2,000, noise-tolerance (tau) was taken as 0, omegas were initialized uniformly as 1. VMD modes were extracted using the Equations 2–4. These features were given as input to the classifiers. Table 6 lists the binary classification performance of the four classifiers for VMD features. K-nearest neighbor classifier correctly classified 60% of the instances from UWS and MCS groups. Its precision and recall were 44.2% and 57.5%, respectively, indicating that it possessed comparatively less capability to identify both positive and negative instances. The F1 score, which combines precision and recall, was 50%. Support vector machine classifier with linear kernel performed with an accuracy of 80%. Decision tree classifier obtained an accuracy of 63.3%. EBT classifier achieved the highest accuracy of 83.3% for 2-group classification, indicating a high percentage of correct classifications. It showed 84.2% precision, 87.5% recall, and an F1-score of 85.8%.

**Table 6:** Binary (UWS vs. MCS) classification results (in %) using 10-fold cross-validation employing all the VMD-based features.

**Classifier**	**Accuracy**	**Precision**	**Recall**	**F1 score**
KNN	60	44.2	57.5	50
Li.SVM	80	77.5	82.5	79.9
DT	63.3	62.5	67.5	64.9
**EBT**	**83.3**	**84.2**	**87.5**	**85.8**

Number of patients: 30. Ensemble bagged tree performs the best with VMD-based features. The bold values indicate the performance obtained by the proposed VMD approach and indicating EBT classifier combined with the proposed approach gives better performance than other classifiers.

Table 7 shows the multiclass performance of various classifiers for features derived from VMD. KNN showed only 46% accuracy with a precision and recall of 32.3% and 45%, respectively. Support vector machine classifier with linear kernel performed with an accuracy of 57%. Decision tree classifier obtained a better accuracy of 73.5%, and a precision of 67.2%, and a recall of 73.3%, showing a good ability to correctly identify positive instances. The F1 score was 70.1%, suggesting a balanced performance. EBT classifier achieved the highest accuracy of 76%. It had a precision of 78.9%, suggesting a low false positive rate, and a recall of 78.3%, implying a good ability to identify positive instances for 3-group classification. The F1 score was 78.6%, indicating a balanced and better performance.

**Table 7:** Multi-class (Coma vs. UWS vs. MCS) classification results (in %) using 10-fold cross-validation employing all the VMD-based features.

**Classifier**	**Accuracy**	**Precision**	**Recall**	**F1 score**
KNN	46	32.3	45	37.6
Li.SVM	57	46.9	56.7	51.3
DT	73.5	67.2	73.3	70.1
**EBT**	**76**	**78.9**	**78.3**	**78.6**

Number of patients: 45. Ensemble bagged tree performs the best with VMD-based features. The bold values indicate the performance obtained by the proposed VMD approach and indicating EBT classifier combined with the proposed approach gives better performance than other classifiers.

### 7.5 Performance comparison between the three approaches

Table 8 lists the improvement in performance with VMD-derived features over the ones derived from the frequency band and raw EEG approaches. Except in the case of KNN multiclass classifier, there is increase in accuracy with VMD-based features over the other two approaches with all the classifiers. With the multiclass classification using EBT classifier, there is a relative improvement of 78.8% in accuracy from the value of 42.5% obtained with frequency band derived features to 76% with VMD-derived features. Similarly, the binary classification accuracy increases from 36.7% to 83.3%, which is a relative improvement of 127%.

**Table 8:** Comparison of percentage increase in accuracy of VMD-derived features over frequency band-derived (FB) and raw EEG-derived (EEG) features, when all the features are employed in each of the approaches.

	**Multiclass classification**		**Binary classification**	
**Classifier**	**FB-features**	**Raw EEG-features**	**FB-features**	**Raw EEG-features**
KNN	33.3	0	63.5	5.8
Li.SVM	26.7	60.6	19.9	9.1
DT	69	83.8	111	18.8
EBT	78.8	67.0	127	38.8

## 8 Statistical analysis: selection of Kruskal-Wallis test

The statistical analysis of the data was conducted using Kruskal-Wallis test, which was selected based on the following considerations. It is a non-parametric technique, which compares the medians of two or more independent groups (Clark et al., 2023). It is essentially a rank-based test and can be used when the assumptions of one-way ANOVA are not met, particularly when the data does not follow a normal distribution. The following are some of the merits of this test, which make it suitable to handle the complexity and of the EEG signal:

- Non-parametric: This test does not presume Gaussian distribution of the analyzed data, making it a preferred choice for skewed or ordinal data.
- Robust to outliers: Compared to parametric tests, Kruskal-Wallis test is less sensitive to outliers since it utilizes the ranks of the data and not their actual values.
- Versatility: It can be applied to more than two independent groups. Thus, it is more versatile than the two-sample Mann-Whitney *U* test.

The above properties of Kruskal-Wallis test make it particularly suitable for EEG data, which often exhibits non-normal distributions and high variability. However, while it tells us whether there is a statistically significant difference between the groups, it does not pinpoint where this distinctness occurs. For the latter, *post-hoc* tests are needed. Further, it presumes that the distributions of the groups are similar in shape. If this assumption is not valid, the results may be questionable. In our case, we use this test to compare the 3 groups of coma, UWS, and MCS. The examination was carried out for every feature derived from the raw EEG approach, frequency bands-based approach, and the VMD-based approach employing a significance level of *p* < 0.05. Following the Kruskal-Wallis test, Dunn's *post-hoc* method was employed to compare multiple groups. The subset of features that showed a significance level of *p* < 0.05 were selected in each of the three approaches to investigate whether they can lead to improved accuracies. Thus, another round of binary and multiclass classifications was conducted using 10-fold cross-validation.

### 8.1 Clinical implications of correlations

**Figure 6:**
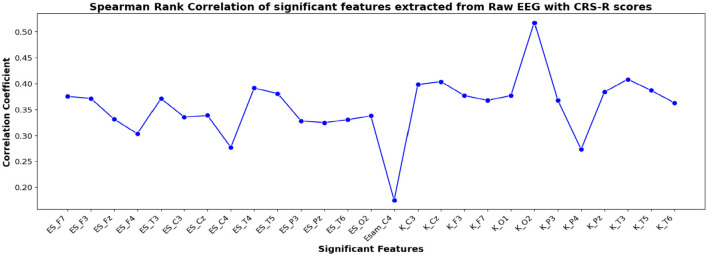
Spearman rank correlation coefficients (y-axis) between the CRS-R scores of 45 patients and each of the chosen feature-electrode combinations (with significance *p* < 0.05) amongst the 4 features extracted from each of the 17 EEG channels (x-axis) of raw EEG.

**Figure 7:**
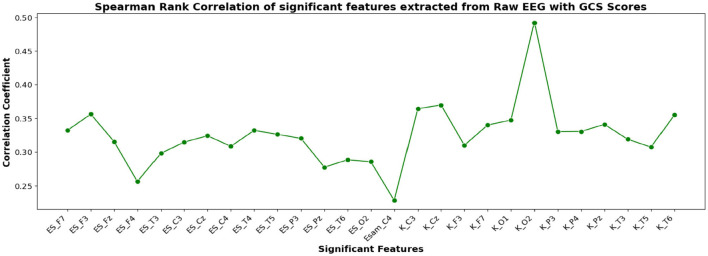
Spearman rank correlation coefficients (y-axis) between the GCS scores of 45 patients and each of the chosen feature-electrode combinations (with significance *p* < 0.05) amongst the 4 features extracted from each of the 17 EEG channels (x-axis).

**Figure 8:**
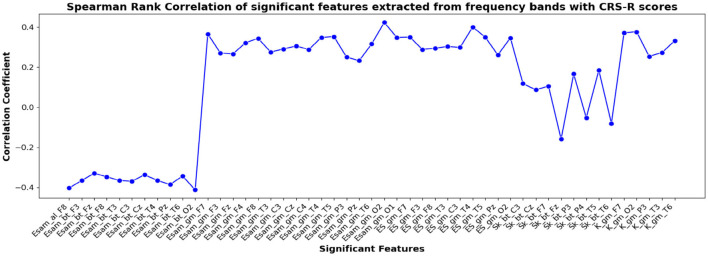
Spearman rank correlation coefficients (y-axis) between the CRS-R scores of 45 patients and each of the chosen feature-electrode combinations (with significance *p* < 0.05) amongst the features extracted from each of the five frequency bands of the 17 channels (x-axis).

**Figure 9:**
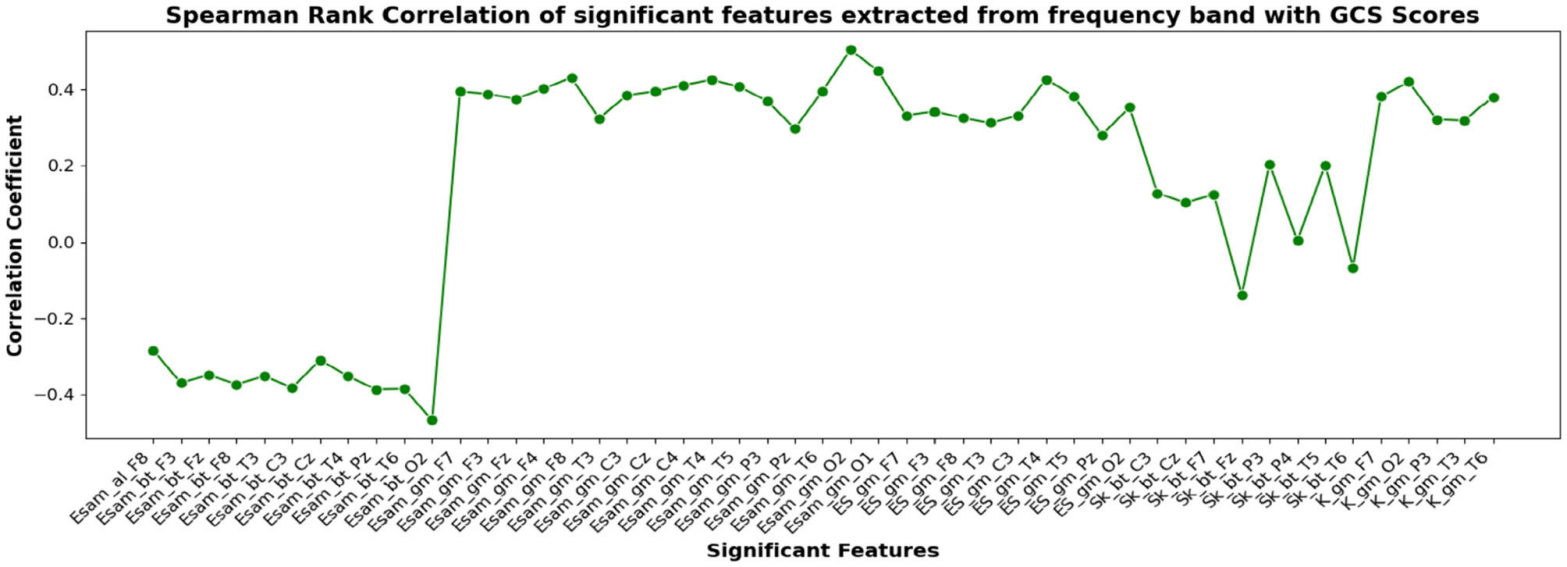
Spearman rank correlation coefficients (y-axis) between the GCS scores of 45 patients and each of the chosen feature-electrode combinations (with significance *p* < 0.05) amongst the features extracted from each of the five frequency bands of the 17 channels (x-axis).

**Figure 10:**
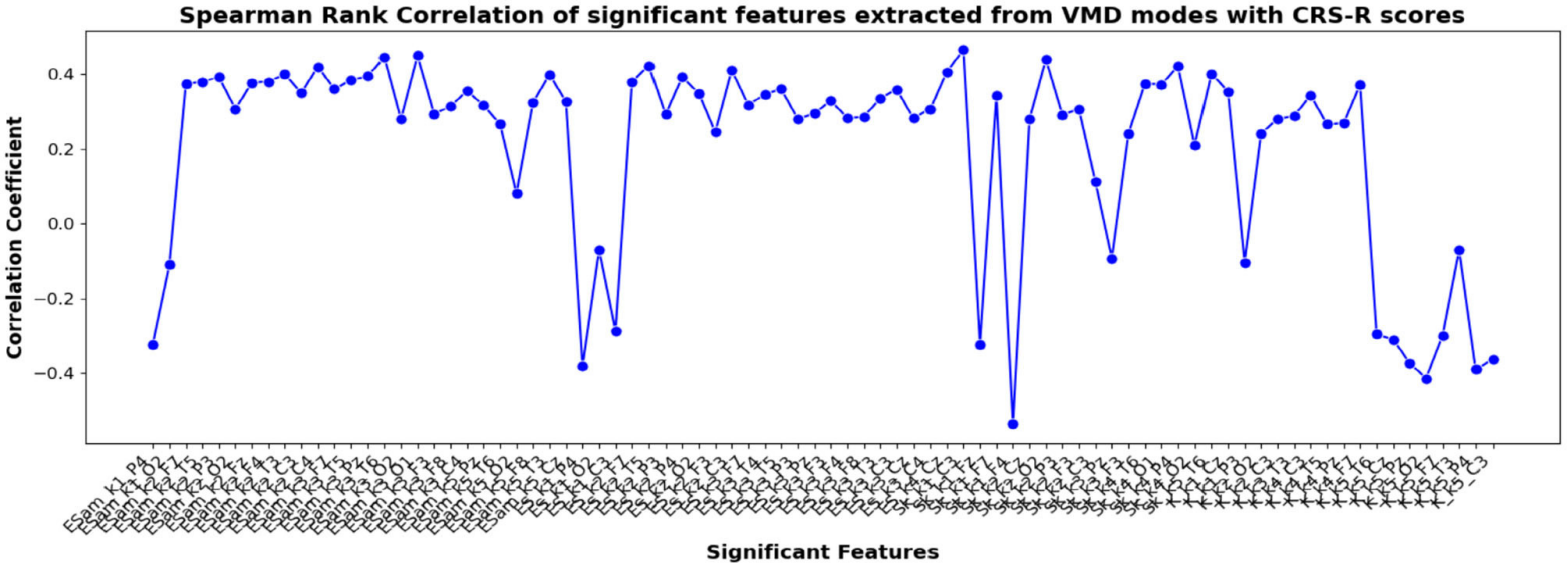
Spearman rank correlation coefficients (y-axis) between the CRS-R scores of 45 patients and each of the chosen feature-electrode combinations (with significance *p* < 0.05) amongst the features extracted from each of the five VMD modes of the 17 channels (x-axis).

**Figure 11:**
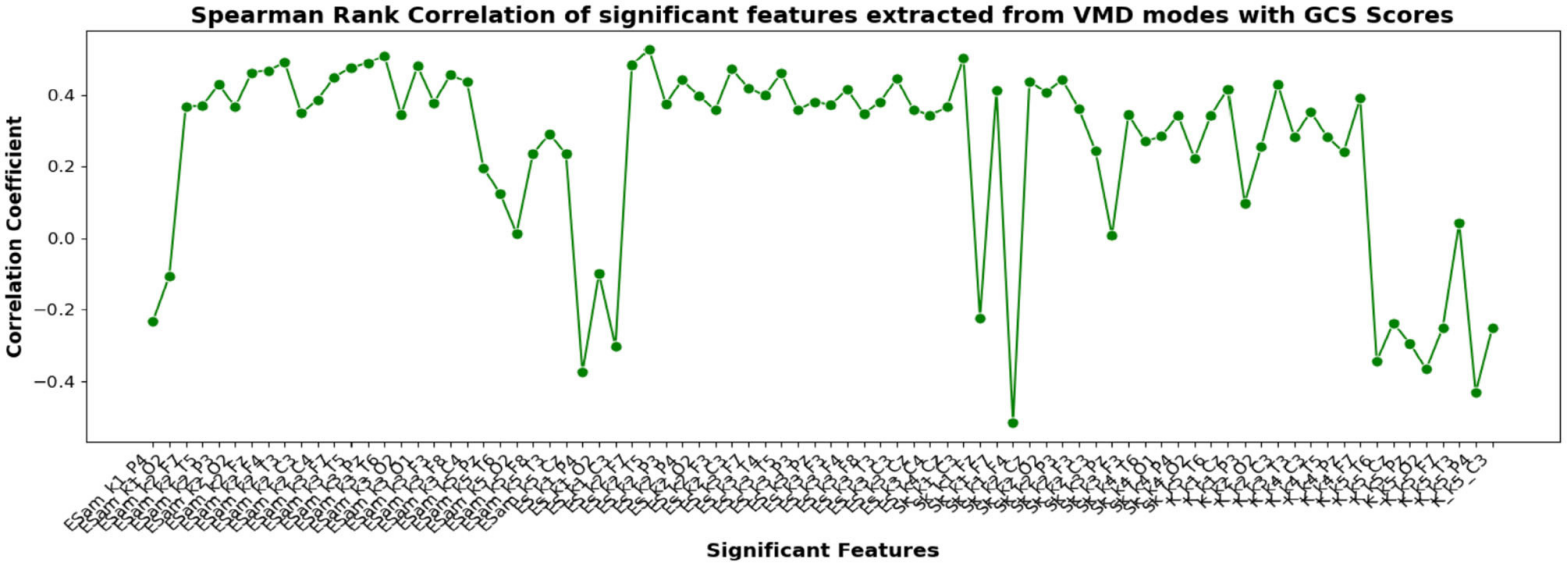
Spearman rank correlation coefficients (y-axis) between the GCS scores of 45 patients and each of the chosen feature-electrode combinations (with significance *p* < 0.05) amongst the features extracted from each of the five VMD modes of the 17 channels (x-axis).

The correlation coefficients of the significant feature-electrode combinations extracted from the raw EEG approach, frequency band-based approach, and VMD approach with CRS-R and GCS scores were estimated using the Spearman Rank correlation method. The correlation coefficients of each of the chosen electrode-feature combinations for the different approaches are shown in Figures 6–11. It can be seen that feature selection is effective in that most of the chosen features have correlation coefficients with absolute values varying between 0.2 and 0.5.

The results obtained for binary and multiclass classification using the reduced set of chosen features are shown for all the approaches in Tables 9, 10, respectively. The results showed that the performance of the classifiers increased when only significant features were considered for classification. The utilization of significant features obtained using Kruskal-Wallis test in classification resulted in enhanced accuracy for all the approaches. The performance of the EBT classifier in the VMD-based approach saw an improvement from 83.3% to 86.7% in effectively distinguishing between UWS and MCS. For multiclass classification using EBT in VMD based approach, the performance increased from 76% to 80.5% with precision and recall of 81.1% and 81.7%. The F1 score improved from 78.6% to 81.4%.

**Table 9:** Binary (UWS vs. MCS) classification results (in %) using 10-fold cross-validation with significant features extracted using Kruskal-Wallis test for the 3 different approaches.

**Binary classification**					
	**Classifier**	**Accuracy**	**Precision**	**Recall**	**F1 score**
Approach 1: features derived from the raw EEG	KNN	56.7	52.5	57.5	54.9
	Li.SVM	50	49.2	52.5	50.8
	DT	43.3	40.8	47.5	43.9
	EBT	53.3	50	60	54.5
Approach 2: features derived from the frequency bands	KNN	70	67.5	67.5	67.5
	Li.SVM	46.7	35.8	47.5	40.8
	DT	56.7	39.2	47.5	43
	EBT	76.7	70.8	77.5	74
Approach 3: features derived from **VMD modes**	KNN	73.3	60.8	72.5	66.1
	Li.SVM	80	68.3	75	71.5
	DT	66.7	63.3	65	64.1
	**EBT**	**86.7**	**83.3**	**85**	**84.1**

Number of patients: 30. The bold values indicate the performance obtained by the proposed VMD approach and indicating EBT classifier combined with the proposed approach gives better performance than other classifiers.

**Table 10:** Multi-class (Coma vs. UWS vs. MCS) classification results (in %) using 10-fold cross-validation with significant features extracted using Kruskal-Wallis test for the 3 different approaches.

**Multiclass classification**					
	**Classifier**	**Accuracy**	**Precision**	**Recall**	**F1 score**
Approach 1: features derived from the raw EEG	KNN	44	37.2	45	40.7
	Li.SVM	32.5	21.6	36.7	27.2
	DT	55	50.3	56.7	53.3
	EBT	50	45.3	51.7	48.3
Approach 2: features derived from the frequency bands	KNN	42	33.9	45	38.7
	Li.SVM	49	38	53.3	44.4
	DT	52	42.8	55	48.1
	EBT	53.5	44.7	56.7	50
Approach 3: features derived from **VMD modes**	KNN	61	53.1	58.3	55.6
	Li.SVM	70.5	67.8	75	71.2
	DT	57.5	50	58.3	53.8
	**EBT**	**80.5**	**81.1**	**81.7**	**81.4**

Number of patients: 45. The bold values indicate the performance obtained by the proposed VMD approach and indicating EBT classifier combined with the proposed approach gives better performance than other classifiers.

EEG being a complex signal, absolute values of correlation of above 0.2 imply that the corresponding EEG-derived features have the potential to indicate improvement in the clinical condition of the patients. So, in addition to the assessment by the therapist, we could explore the direct use of these features as possible indicators of the clinical condition of the patients, and their credibility for assessing the improvement in the patients as an outcome of the treatment strategy determined. If successful, such EEG-based features will be valuable additions for objective clinical evaluation of the DOC patients.

### 8.2 Improvement in the performance of the classifiers due to feature selection

Table 11 shows the advantage gained by the rejection of VMD-based features which are not significant as determined by the Kruskal-Wallis test. The Table reveals that the accuracy of all the classifiers increases due to feature selection, except DT in the multiclass scenario, and SVM in the binary scenario. By taking the performance of the EBT classifier beyond 80% in multiclass classification, this establishes the importance of feature selection in such machine learning problems. The inclusion of features that are not statistically significant confuses the classifier during training, leading to lower recognition performance. Thus, eliminating such features has the potential to improve the model's ability to generalize and perform equally well on new, unseen data.

**Table 11:** Improvement in accuracy (%) of different classifiers due to the selection of features shown to be statistically significant by Kruskal-Wallis test: VMD-derived features.

	**Multiclass classification**			**Binary classification**		
**Classifier**	**All features**	**Sel. features**	**IA**	**All features**	**Sel. features**	**IA**
KNN	46.0	61.0	15.0	60.0	73.3	13.3
Li.SVM	57.0	70.5	13.5	80.0	80.0	0
DT	73.5	57.5	-16.0	63.3	66.7	3.4
EBT	76.0	80.5	4.5	83.3	86.7	3.4

IA, absolute improvement in accuracy.

### 8.3 Performance comparison between the three approaches post feature selection

Table 12 compares the performance of the three approaches after feature selection based on the significance scores given by the Kruskal-Wallis test. Once again, the accuracy achieved by VMD-based features is higher than those of the other two approaches, irrespective of the classifier employed. Thus, even with the best subset of features chosen for each approach, VMD-derived features achieved a relative improvement of 50.5% and 61% in performance over frequency-based and raw-EEG-based features, respectively, in the multiclass classification scenario. For the binary classification scenario, the corresponding figures are 13% and 62.7%, respectively.

**Table 12:** Comparison of percentage increase in accuracy of VMD over frequency band-derived (FB) and raw EEG-derived (EEG) features after feature selection based on Kruskal Wallis test.

	**Multiclass classification**		**Binary classification**	
**Classifier**	**FB-features**	**Raw EEG-features**	**FB-features**	**Raw EEG-features**
KNN	45.2	38.6	4.7	29.3
Li.SVM	43.9	116.9	71.3	60
DT	10.6	4.5	17.6	54
EBT	50.5	61	13	62.7

## 9 Discussion

The proposed work investigated the application of variational mode decomposition as the initial processing method before feature extraction for resting state EEG signals of DOC patients. It aimed to apply machine learning models for distinguishing DOC patients without relying solely on behavioral assessment methods.

It also aimed to compare the VMD-based features with those derived from commonly used frequency bands. The study showed that VMD-derived features gave better results. Tables 8, 12 present the percentage increase in accuracy achieved by the VMD-derived features over those derived from both the frequency bands and the raw EEG signals with all the features extracted and with significant features obtained using statistical analysis test, respectively.

Table 13 compares the performance of various existing works in EEG-based classification of DOC classes, each employing distinct features and classifiers. The studies evaluated include those by Höller et al. (2014), Sitt et al. (2014), Chennu et al. (2017), Engemann et al. (2018), and Di Gregorio et al. (2022). Höller et al. (2014) focused on directed transfer function features, employing Li.SVM classifiers for differentiating between patients in MCS and UWS states with high accuracy, reaching 82% for MCS vs. UWS and 92% for healthy controls (HC) vs. MCS. Sitt et al. (2014) utilized a combination of weighted symbolic mutual information (wSMI), permutation entropy (PE), spectral entropy, and KCC features with SVM classifiers, achieving an accuracy of 89% for the binary classification of MCS and UWS patients. The study provided a detailed description of various features extracted from the EEG signals. Engemann et al. (2018) employed features such as normalized alpha-band power, PE, wSMI, and complexity measures to discriminate between MCS and UWS patients, achieving an unspecified accuracy with SVM classifiers.

**Table 13:** Comparison of performance with existing work.

**References**	**Features**	**No. of subjects**	**Classifier**	**Accuracy (%)**	**Precision (%)**
Höller et al. (2014)	Directed transfer function generalized partial directed coherence partial coherence	MCS 22, UWS 27, Controls 23	Li.SVM	MCS vs. UWS: 82	-
				HC vs. MCS: 92	-
				HC vs. UWS: 92.6	-
Sitt et al. (2014)	wSMI, PE, SpecE, KCC	MCS 68, UWS 75	SVM	89	-
Engemann et al. (2015)	Normalized alpha-band power, PE, wSMI, complexity measure	MCS 69, UWS 76	SVM	-	-
Chennu et al. (2017)	Spectral power, dwPLI, graph-theoretic metrics	UWS 23, MCS- 17, MCS + 49, controls 26	Rb.SVM	79	-
Di Gregorio et al. (2022)	Dominant frequency, PCoh MI, PCoh	DOC 33	LDA	TBI: 80	77.7
				Non-TBI: 83.3	85.7
**Proposed Method** (features selected by Kruskal-Wallis test)	VMD mode based features: kurtosis, skewness, spectral entropy, sample entropy	Coma 15, UWS 15, MCS 15	Ensemble bagged tree	UWS vs. MCS: **86.7**	**83.3**
				Coma vs. UWS vs. MCS: **80.5**	**81.1**

The bold values indicate the performance obtained by the proposed VMD approach and indicating EBT classifier combined with the proposed approach gives better performance than other classifiers. HC, healthy controls; wSMI, weighted symbolic mutual information; PE, permutation entropy; KCC, Kolmogorov Chaitin complexity; dwPLI, debiased weighted phase lag index; MI, mutual information; PCoh, partial coherence; TBI, traumatic brain injury.

Chennu et al. (2017) utilized spectral power, debiased weighted phase lag index (dwPLI), and graph-theoretic metrics with an Rb.SVM classifier to distinguish between different states (UWS, MCS–, MCS+, and controls), achieving an accuracy of 79%. Di Gregorio et al. (2022) incorporated features like mutual information (MI), dominant frequency, and partial coherence (PCoh) with LDA classifiers to differentiate between traumatic brain injury (TBI) and non-TBI patients, achieving varying accuracies for different TBI subgroups; without specifically classifying the DOC categories. All the above studies considered only binary classification between MCS and UWS, and coma patients were not included in any of the studies.

The proposed method in this study employs VMD mode-based features and an ensemble bagged tree classifier for classifying coma, UWS, and MCS patients. The results showcase a promising accuracy of 86.7% in distinguishing between UWS and MCS states and 80.5% accuracy in distinguishing among coma, UWS, and MCS states. This suggests that the VMD mode-based features, in conjunction with the EBT classifier, offer competitive performance compared to existing methods, especially in the challenging task of discriminating among different levels of consciousness in patients.

When compared to the frequency band-based features, an advantage of VMD-based features of resting-state EEG of DOC patients is its ability to capture transients and non-stationarities. Patients with DOC often exhibit intermittent moments of consciousness or changes in their neurological state. Traditional frequency bands might struggle to capture these nuanced shifts, whereas VMD can identify and isolate these dynamic changes in neural activity. This capability is particularly relevant in clinical contexts, where monitoring the patient's responsiveness and potential for recovery is of utmost importance. Furthermore, VMD can provide enhanced spatial localization of neural activity by decomposing EEG signals into spatially independent modes. This spatial information can be invaluable for pinpointing the source of abnormal brain activity in DOC patients and potentially guiding targeted interventions such as neuromodulation or neurofeedback. Another advantage of VMD is its adaptability to various EEG data acquisition setups and patient-specific characteristics. Since VMD does not rely on predefined frequency bands, it can be applied across different EEG protocols and it adapts to the individual patient's unique neural dynamics. This flexibility makes VMD a promising tool for the classification of DOC. The ability of the VMD method to analyze complex EEG signals of various conditions (medical and non-medical) has been explored by researchers and found that it performed better than other signal processing methods. From the literature review, it was found that VMD has not been applied for analyzing DOC patients. Thus this study fills up the gap in the literature on DOC analysis. It is important to note that while VMD offers numerous advantages, it is not a replacement for traditional EEG frequency band analysis. Instead, these two approaches can complement each other. Researchers and clinicians could use traditional frequency bands to assess the patient's overall level of consciousness and cognitive functioning, while VMD can provide a dynamic picture of neural activity.

### 9.1 Implications of the findings for clinical practice and future research

Since the results we have obtained are the best in the literature for the 3-class classification problem, our approach is encouraging. The reliability of the performance of the model must be established by using it in a clinical setup, while simultaneously collecting more patient data. This will be attempted in NIMHANS, Bangalore so that the effectiveness of the model on new patients can be studied, leading to improvement of the model over time. Better classification of the nature of DOC will definitely lead to better treatment strategies. However, more comprehensive data must be collected that includes comparable number of patients from different aetiologies, and other features can be experimented with to enhance the performance much further. It will be ideal if different clinical groups working across different countries come together and create a master database of a significant number of patients covering the various causes of DOC. This will lead to more coordinated and effective research and we can expect that this will lead to a classification scheme that performs better on new, unseen data.

Further, better classification will also lead to better assessment of treatment strategies, since if a UWS patient improves faster and becomes MCS, which is accurately reflected by the classifier model, it will validate both the model and the treatment strategy.

### 9.2 Limitations of our work

However, the challenges in utilizing VMD for EEG signal analysis arise from the necessity to choose parameters, such as the number of modes and regularization parameters. In the study, the number of modes has been selected as 5 based on literature which is a limitation of this study. Inaccurate parameter selection may yield decomposition results that are not reliable. Furthermore, VMD presupposes that the original signal is a linear combination of modes with distinct frequency components. In the context of EEG analysis, VMD demonstrates heightened computational complexity, especially for lengthier EEG signal recordings or datasets with high dimensions. This increased complexity can render the analysis time-consuming, restricting its real-time applicability and posing challenges for large-scale studies.

#### 9.2.1 Effect of sample size and distribution on the validity of results

The effect of size and distribution of samples on the reliability of the findings of machine learning models is an important topic in machine learning research (Rajput et al., 2023). Below, we give an overview of this issue.

- How the data is balanced across different classes and how it is split into training, validation, and testing sets impacts the acceptability of the models concerning their evaluation, bias, and variance.
- If the model is too simple and does not fully capture the underlying structure of the data, we say that sample distribution is poor, resulting in high bias and low variance.
- If the model is trained and tested on representative and diverse samples of the data, we say that the sample distribution is good, ensuring low bias, and low variance.
- The number of data points used to train and test a machine learning model impacts its validity in terms of accuracy, reliability, and generalization.
- A low sample size may lead to overfitting, which implies that the model learns the patterns specific to the training data including any noise present, and hence may fail to do well on new data.
- A high sample size may increase the robustness and classification accuracy of the model; however, it may not improve significantly after a certain size, depending on the variability, and complexity of the data.
- Various validation methods, namely k-fold cross-validation, nested cross-validation, and train/test split, are used to determine the model's performance on unseen data and avoid both underfitting and overfitting.

Obtaining an equal number of coma patients and ensuring the hemodynamic stability of DOC patients proved to be challenging. The reproducibility and generalizability of findings hinge on the rigor of inclusion/exclusion criteria, the behavioral scores assigned to patients, and the processing steps employed during analysis. Notably, the outcome analysis may have been influenced by recruitment bias stemming from difficulties in conducting EEGs within the ICU setting, leading to the inclusion of a more diverse patient population.

## 10 Conclusion

This paper presents an exploratory study on the application of VMD for classifying patients with disorders of consciousness into different categories, namely coma, UWS, and MCS. In the proposed approach, four features namely sample entropy, spectral entropy, kurtosis, and skewness were derived from three distinctly processed EEG signals. The analysis was performed on the dataset collected from NIMHANS. The results showed that VMD-derived features distinguished the three classes more effectively than the frequency band-derived and raw EEG-derived ones. The accuracy of 80.5% we have obtained is the best in the literature for the 3-class classification problem. It is also observed that the ensemble bagged tree classifier using VMD features performed better in classifying different DOC cohorts than KNN, DT, and SVM classifiers. Thus the study focused on the ability to classify DOC patients based on the features extracted from VMD modes. However, since the dataset is considerably small and the classification of DOC patients based on machine learning methods is an emerging area of research, the results obtained cannot be generalized for DOC patients as a whole. Future research will center on improving the VMD methodology through specific modifications to address challenges in determining the number of modes and regularization parameters. Moreover, there will be a focus on integrating an adaptive filter into the extraction of frequency bands from EEG signals. This strategic integration aims to resolve issues such as edge effects, lack of adaptability to signal changes, suboptimal performance under varying EEG signal properties, and the inability to effectively capture non-stationary signal changes.

Despite these limitations, the suggested approach illustrates how the integration of machine learning and signal decomposition techniques, such as VMD, can assist clinicians in classifying individuals with different disorders of consciousness.

## Data availability statement

The datasets presented in this article are not readily available because to maintain the confidentiality of patients with DOC, the dataset is not made available. Requests to access the datasets should be directed to SRam, subasree.ramakrishnan@gmail.com.

## Ethics statement

The studies involving humans were approved by Institute Ethics Committee (Basic Sciences & Neurosciences), NIMHANS, Bangalore, India. The studies were conducted in accordance with the local legislation and institutional requirements. The participants provided their written informed consent to participate in this study.

## Author contributions

SRav: Conceptualization, Formal analysis, Investigation, Methodology, Software, Writing – original draft, Writing – review & editing. RK: Methodology, Resources, Software, Supervision, Validation, Writing – review & editing. SK: Writing – review & editing. JS: Writing – review & editing. MF: Resources, Writing – review & editing. RC: Resources, Writing – review & editing. SB: Resources, Writing – review & editing. VB: Resources, Writing – review & editing. AR: Methodology, Software, Supervision, Validation, Writing – review & editing. SRam: Conceptualization, Investigation, Methodology, Resources, Supervision, Validation, Writing – review & editing. SK: Project administration, Supervision, Validation, Writing – review & editing.
